# Optimizing imaging in suspected appendicitis (OPTIMAP-study): A multicenter diagnostic accuracy study of MRI in patients with suspected acute appendicitis. *Study Protocol*

**DOI:** 10.1186/1471-227X-10-19

**Published:** 2010-10-20

**Authors:** Marjolein MN Leeuwenburgh, Wytze Laméris, Adrienne van Randen, Patrick MM Bossuyt, Marja A Boermeester, Jaap Stoker

**Affiliations:** 1 Department of Surgery, Academic Medical Center, University of Amsterdam, Meibergdreef 9, Amsterdam, the Netherlands; 2 Department of Radiology, Academic Medical Center, University of Amsterdam, Meibergdreef 9, Amsterdam, the Netherlands; 3 Department of Clinical Epidemiology Biostatistics and Bioinformatics, Academic Medical Center, University of Amsterdam, Meibergdreef 9, Amsterdam, the Netherlands

## Abstract

**Background:**

In patients with clinically suspected appendicitis, imaging is needed to substantiate the clinical diagnosis. Imaging accuracy of ultrasonography (US) is suboptimal, while the most accurate technique (CT) is associated with cancer related deaths through exposure to ionizing radiation. MRI is a potential replacement, without associated ionizing radiation and no need for contrast medium administration. If MRI is proven to be sufficiently accurate, it could be introduced in the diagnostic pathway of patients with suspected appendicitis, increasing diagnostic accuracy and improving clinical outcomes, without the risk of radiation induced cancer or iodinated contrast medium-related drawbacks. The multicenter OPTIMAP study was designed to estimate the diagnostic accuracy of MRI in patients with suspected acute appendicitis in the general population.

**Methods/Design:**

Eligible for this study are consecutive patients presenting with clinically suspected appendicitis at the emergency department in six centers. All patients will undergo imaging according to the Dutch guideline for acute appendicitis: initial ultrasonography in all and subsequent CT whenever US does not confirm acute appendicitis. Then MRI is performed in all patients, but the results are not used for patient management. A final diagnosis assigned by an expert panel, based on all available information including 3-months follow-up, except MRI findings, is used as the reference standard in estimating accuracy. We will calculate the sensitivity, specificity, predictive values and inter-observer agreement of MRI, and aim to include 230 patients. Patient acceptance and total imaging costs will also be evaluated.

**Discussion:**

If MRI is found to be sufficiently accurate, it could replace CT in some or all patients. This will limit or obviate the ionizing radiation exposure associated risk of cancer induction and contrast medium induced nephropathy with CT, preventing the burden and the direct and indirect costs associated with treatment. Based on the high intrinsic contrast resolution of MRI, one might envision higher accuracy rates for MRI than for CT. If so, MRI could further decrease the number of unnecessary appendectomies and the number of missed appendicitis cases.

**Trial registration:**

NTR2148

## Background

Acute appendicitis is one of the most common reasons for acute abdomen [1]. Diagnosis based on clinical evaluation only is difficult and results in high negative appendectomy rates and missed diagnoses [2,3]. Negative appendectomies increase mortality, prolong hospital stay, and increase the risk of infectious complications [4]. Appendicitis is missed in approximately 12% of patients, increasing the risk of perforated appendicitis, peritonitis, abscesses and leading to a two to tenfold increased mortality rate [5-7].

The use of ultrasonography (US) and computed tomography (CT) to support clinical diagnosis is widespread [8]. US has considerable accuracy limitations, as it generates too many false negative results. Although CT is more accurate, it fails in 12% of patients and results in considerable ionizing radiation exposure in often young individuals. This ionizing radiation exposure is associated with the risk of cancer induction and cancer related death [9]. Iodinated contrast medium administration may also induce nephropathy or aggravate existing nephropathy.

MRI is a potential replacement for CT, without associated ionizing radiation and contrast medium administration. If proven to be sufficiently accurate, MRI could be introduced in the diagnostic pathway of patients with suspected appendicitis, increasing diagnostic accuracy and improving clinical outcome, without the risk of radiation induced cancer or iodinated contrast medium-related drawbacks.

Available studies on the diagnostic accuracy of MRI are limited in size, single center and primarily included selected patients - often pregnant women - with a different spectrum and prevalence of disease compared to the general population. In these studies 10% of patients had appendicitis, substantially lower than the usual 60%. Experience with newer MRI techniques that may boost its accuracy, such as diffusion-weighted imaging (DWI), is even more limited.

These results do not justify introducing MRI as first line imaging technique in patients suspected for acute appendicitis yet. To evaluate the potential of MRI as an alternative imaging method in patients with suspected appendicitis, we need a sufficiently powered study in unselected patients. The present study will allow us to estimate the accuracy of MRI in unselected patients and to compare with that of CT. This may help us to identify the optimal diagnostic strategy, selecting from available imaging modalities, aiming at high diagnostic accuracy without compromising health care while minimizing radiation exposure.

## Methods/Design

### Study objectives

The OPTimizing IMaging in suspected APpendicitis (OPTIMAP) study aims to assess the diagnostic accuracy of MRI in unselected patients presenting with suspected acute appendicitis, and to estimate its costs, inter-observer agreement and patient acceptance.

### Study design

OPTIMAP is a multicenter diagnostic accuracy study of MRI in a consecutive series of adult patients with clinically suspected acute appendicitis. Consenting patients will undergo initial ultrasonography followed by CT in all cases in which US does not confirm the suspected appendicitis, which is the strategy specified in the Dutch guideline for suspected acute appendicitis. Additionally, all patients undergo MRI, with the MRI reader blinded from the results of the other imaging methods. A final diagnosis assigned by an expert panel based on all available data (except MRI) after 3 months follow-up will act as the reference standard in estimating accuracy.

### Study population

Eligible are consecutive adult patients, 18 years or older, with clinically suspected acute appendicitis presenting at the emergency department. Excluded are pregnant patients, patients with contraindications for MRI scanning and critically ill patients that need intensive vital organ function monitoring for life-support.

We will recruit patients in one university hospital (Academic Medical Center, Amsterdam) and five large teaching hospitals in the Netherlands (Medical Center Alkmaar; Antonius Hospital, Nieuwegein; Sint Lucas Andreas Hospital, Amsterdam; Gelre Hospital, Apeldoorn; Kennemer Gasthuis, Haarlem). Treating physicians in the emergency department will identify eligible patients based on medical history, physical and laboratory examination prior to imaging. Eligible patients will be informed about the study and invited to participate.

### Ethical considerations

The OPTIMAP study will be conducted according to the principles of the Helsinki Declaration and in accordance with the Medical Research Involving Human Subjects Act (WMO) and other European guidelines, regulations and acts. The Medical Ethical Committee of the Academic Medical Center in Amsterdam approved our study protocol. All participating hospitals gave their consent after assessment of local feasibility. Only patients who give written informed consent will be included in the study.

### Standard care

The findings of clinical assessment, the clinical diagnosis and possible alternative diagnoses, and the level of confidence (certainty) of the clinical diagnosis of acute appendicitis will be prospectively documented by the treating physician in an on line case record form (CRF). Subsequently a staff radiologist or radiological resident will perform an ultrasonography (US). This US concerns a complete examination of the abdomen, including the use of the graded compression technique. In case of a non diagnostic US, an abdominal computed tomography (CT) of the complete abdomen will be performed. All CT scans will be performed using a multi-detector row 4, 16 or 64 slice CT scanner (4-slice SOMATOM Volume Zoom, 16-slice SOMATOM sensation, Siemens Medical Systems, Forchheim, Germany; 16-slice MX 8000, 64-slice Brilliance, Philips Medical Systems, Best, The Netherlands; 64-slice Aquilion, Toshiba Medical Systems, Tokyo, Japan) and intravenous contrast medium. No oral or rectal contrast medium is routinely administrated. The radiologist will record imaging features of the appendix, presence or absence of appendicitis, level of confidence of the diagnosis, and possible alternative diagnoses separately in our online CRF for US or CT.

### MRI examination

Consenting patients will undergo MRI at 1.5 T (MAGNETOM Avanto 1,5 T MRI, Siemens Medical Systems, Forchheim, Germany; Intera 1.5 T MRI, Philips Medical Systems, Best, The Netherlands) within two hours of admission to the emergency department. The MRI examination will comprise breath hold axial and coronal T2 weighted sequences (HASTE: slice thickness 6 mm, FOV 400 mm, TR1500 ms, TE 90 ms, 256 × 256 matrix, flip angle 170; HASTE SPAIR: slice thickness 6 mm, FOV 400 MM, TR 1400 ms, TE 93 ms, 256 × 256 matrix, flip angle 160) and free breathing axial and coronal diffusion weighted sequences (DWI: slice thickness 6 mm, FOV 400 mm, TR 3900 ms, TE 75 ms, B-values 50 - 400 - 800, 192 × 192 matrix). A pilot study in one of the participating institutions has indicated the potential of DWI for acute appendicitis (unpublished data). No intravenous contrast medium is administrated. In-room time will be approximately 15 minutes. In two hospitals (AMC, MCA) MRI examinations will be performed between 8 AM and 11 PM, in the other hospitals during office hours.

### MRI interpretation

All MR scans will be prospectively read by two independent radiologists, blinded for each other's findings, US and CT results. These selected radiologists will be trained to adequately appraise the MR scan for presence or absence of appendicitis. Their training consists of 102 abdominal MR scans in patients presenting previously with clinically suspected appendicitis in one of the participating institutions (Medical Center Alkmaar). The MRI observers will document imaging findings in the on line CRF as described earlier for US and CT. Afterwards; all MRI examinations will be scrutinized by central reading by a MRI expert committee with the same clinical information as the initial MRI readers to establish a reference of optimal MRI accuracy for comparison with clinical practice MRI accuracy.

### Patient management

Patients will be managed based on the US and CT findings. MRI will not be used for management, except in equivocal findings at US and CT, or in case of other clinically important findings at MRI that were undetected at US and CT.

### Reference standard

**Figure 1:**
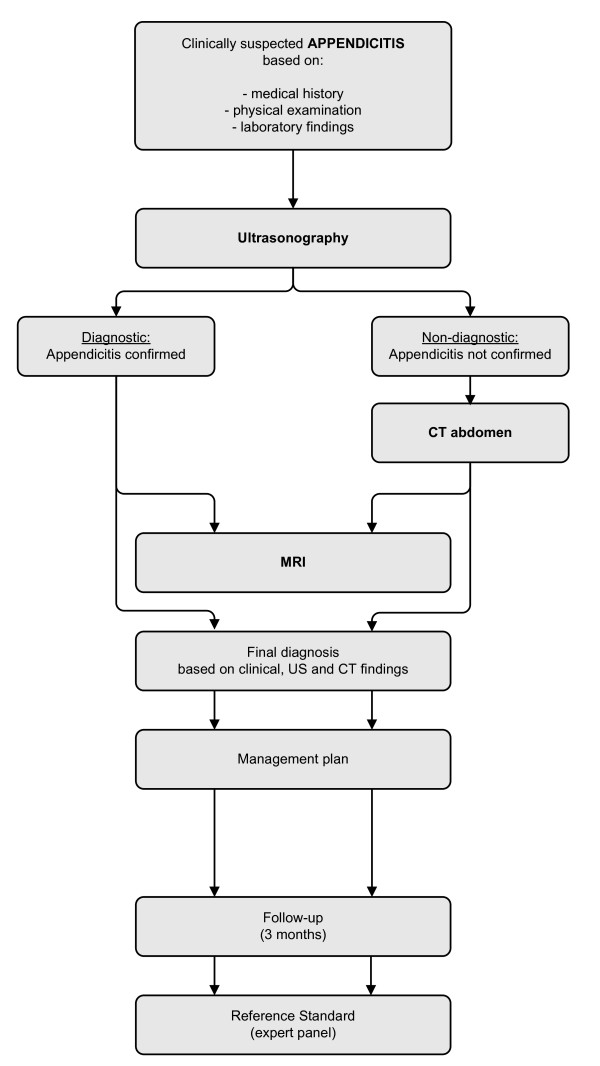
The OPTIMAP study flowchart.

An expert panel consisting of two surgeons and a radiologist will assign a final diagnosis after a follow-up period of 3 months, based on all available information: clinical information, imaging findings (except MRI findings), surgery, pathology and follow up. General practitioners will be contacted to assess whether patients had an appendectomy in another hospital, or an alternative diagnosis assigned. The flowchart in figure 1 demonstrates the complete clinical pathway of included patients in the OPTIMAP study.

### Data Analysis

Data analysis primarily will focus on the diagnostic accuracy of MRI in correctly identifying patients with appendicitis. Sensitivity, specificity, positive and negative predictive value of MRI in detecting acute appendicitis will be calculated with corresponding 95% confidence intervals, by comparing the results of MRI, as read by trained radiologists and the MRI expert panel, with the final diagnosis assigned by the expert panel. In addition, the accuracy of the following scenarios will be estimated: (1) Clinical evaluation without imaging, (2) US in all patients followed by CT after a non diagnostic US, (2) US only, (3) MRI only, (4) US followed by MRI after a non diagnostic US. A gain in diagnostic value of strategies using two tests will be evaluated using the likelihood ratio based method proposed by McAskill and colleagues [10]. Next, we will evaluate the diagnostic performance of stratified imaging strategies taking into account patient characteristics (e.g. age, gender) and presentation features (e.g. duration of complaints). We will also investigate accuracy modifiers, such as body mass index and gender, which are known to influence the diagnostic performance of some imaging modalities.

For the cost evaluation, we will estimate and compare the total imaging costs for each imaging strategy. Standard unit prices will be used for all imaging modalities. Total imaging costs in multi-modality strategies will be driven by the positivity rate of the first imaging procedure.

To evaluate inter-observer agreement we will calculate percentage agreement and kappa values for the diagnosis of appendicitis in MRI between observer 1 en 2 (clinical practice reading) en central reading. Effects of reader experience will be evaluated by comparing the accuracy of local reading (single observer) to the accuracy of the expert central reading.

We will evaluate patient acceptance of MRI in comparison to standard imaging practice as documented in the Dutch guidelines. For each examination participants are invited to rate their experience (including burden, discomfort and pain) using five-point Likert scales (none, mild, moderate, severe, extreme). Differences between US, CT and MRI will be tested for statistical significance.

### Sample size calculation

We anticipate an MRI sensitivity of 90% and specificity of 95%, based on accuracy results in published series of primarily pregnant women [11]. Approximately 60% of patients with suspected appendicitis are expected to have a final diagnosis of appendicitis, based on the findings in the OPTIMA trial, which had comparable inclusion criteria and ran in similar hospitals [12]. To obtain sensitivity and specificity estimates with 95% confidence intervals not exceeding 10%, a study group of 230 patients is required. Of the 230 patients, 138 are anticipated to have acute appendicitis (60%), while MRI will correctly identify appendicitis in 124 (sensitivity 90%; 95% CI 84% to 94%) and correctly exclude appendicitis in 95 (specificity 95%; 95% CI 88% to 98%).

## Rationale for design

It is widely recognized that imaging and other medical tests should be evaluated based on their ability to improve patient outcome or to reduce costs [13]. It is also acknowledged that evaluations of tests benefit from a phased approach, where an assessment of reproducibility and diagnostic validity precede evaluations of overall clinical utility and resource use [14].

For these reasons we decided that an accuracy study, evaluating MRI next to the current best imaging strategy was in place. This will allow us to explore the likely utility of MRI in patients with suspected appendicitis, and to model various imaging scenarios with respect to their ability to identify patients with appendicitis while minimizing imaging costs and radiation exposure. When sufficient evidence has become available about the accuracy of MRI, a study with initial US and randomization for CT or MRI in inconclusive cases can be considered as next research step.

At present MRI is not a routine examination for acute abdomen in general and in suspected acute appendicitis in particular. A pilot study of 70 patients in Alkmaar Medical Center showed that performing MRI in patients with acute abdomen is very well feasible, also after office hours. In the present study two of six participating hospitals will perform MRI outside office hours. Different time windows of inclusion will most likely not be a source of bias. In the OPTIMA study the prevalence of appendicitis was independent of time of presentation (60% during vs. 59% after office hours) [12]. The order of CT and MRI will vary and depend which investigation can be scheduled first. Hereby the imaging delay is minimized. When evaluating patient acceptance (a secondary outcome parameter), the order of investigations can be adjusted for in the analysis.

Several studies, including two RCT's, have showed that the routine use of imaging has a positive effect on patient outcomes in patients with suspected appendicitis [15,16]. The patient population studied in this proposal is identical to the population for which the Dutch guideline has been developed. As in daily practice, patients with a very low suspicion for appendicitis - in whom imaging is not considered required for excluding acute appendicitis - will not be included; these patients will be scheduled for re-evaluation and not for imaging.

The sample size is of this study allows for subgroup analysis of MRI accuracy in what is probably the most important subgroup of patients: women of childbearing age. Of the 230 patients, approximately 130 patients will be female, of which the majority is expected to be of childbearing age.

## Discussion

Abdominal MRI has often been associated with lengthy examination times, which would make it less appropriate for evaluating acute appendicitis. Yet examination time should no longer be a hurdle: with present-day hardware and software, imaging protocols with 15 minutes in-room time suffice for evaluating a patient with suspected appendicitis. MRI is already used for other acute primarily neurological conditions, such as imminent paraplegia. With limited requirements for room time, the availability of MRI for evaluating acute conditions can be expanded to include acute appendicitis, as is already possible in the institutions participating in this study. Our study group is performing a national survey, to evaluate MRI availability for acute diagnosis and identify potential hurdles for the introduction of acute MRI at a national level.

The American College of Radiology has published a consensus document on appropriateness criteria for imaging evaluation of patients with acute pain in the right lower quadrant. The consensus finds CT the most appropriate for these patients [17]. Recently we have published the results of a preceding study in patients with acute abdominal pain, showing that initial US in all and CT in case of negative or inconclusive US was the optimal diagnostic imaging strategy to detect urgent disease [12]. The new Dutch acute appendicitis guidelines have been completed and became effective in March 2010. The imaging proposed in that guideline is the routine strategy in our study protocol, i.e. US in all and CT in negative or inconclusive US cases.

This study aims to determine the optimal diagnostic strategy for patients with suspected acute appendicitis in the emergency department. If MRI is found to be sufficiently accurate, it could replace CT in some or all patients. This will limit CT related cancer induction and death and contrast medium induced nephropathy, preventing the burden and the direct and indirect costs associated with treatment. Radiation exposure of CT is especially a concern in children, pregnant patients, and adults <50 year, but not negligible in individuals ≥ 50 year. Seventy-five percent of adult patients with suspected appendicitis were < 50 year in the OPTIMA study and this proportion will be similar in this study proposal [12]. Until now MRI has almost exclusively been studied in children and pregnant patients [18]. Apart from the risk of cancer induction, CT is associated with the risk of renal insufficiency. Intravenous contrast medium aggravates existing renal insufficiency and induces renal insufficiency in those with marginal renal function [19]. Approximately 60% of patients are not aware of their (imminent) renal insufficiency. The prevalence of (imminent) renal insufficiency increases with age [20]. For MRI no intravenous contrast medium is needed, obviating this risk. MRI can be beneficial for all adult patients irrespective of age. Studying MRI in all adult patients is therefore important.

Based on the high intrinsic contrast resolution of MRI, one might envision higher accuracy rates for MRI than CT, but this needs to be substantiated in this study. If so, MRI could further decrease the number of unnecessary appendectomies and the number of missed appendicitis cases.

## Conclusion

The present work up in adult patients suspected for appendicitis has substantial shortcomings (e.g. proportion negative appendectomies). The most accurate technique - CT - is associated with radiation burden and renal insufficiency.

MRI is a potential valuable technique in all adult patients as it lacks the risks associated with CT and has an accuracy that is presumably comparable or possible higher than CT. Until now, the accuracy of MRI has not been studied in non pregnant adults except in studies limited in size [21]. Therefore more data are needed before further steps (e.g. RCT) can be made. This prospective multi-center study (Trial registration: NTR2148) will provide this information including accuracy, reproducibility, patient acceptance and imaging costs. Scenario analyses will allow us to compare several strategies.

## Prospective

The OPTIMAP study inclusion started in March 2010, results are expected in 2011.

## Competing interests

The authors declare that they have no competing interests.

## Authors' contributions

ML/WL/AR/PB/MB/JS. 1) have made substantial contributions to conception and design 2) have been involved in drafting the manuscript or revising it critically for important intellectual content 3) have given final approval of the version to be published.

Each author has participated sufficiently in the work to take public responsibility for appropriate portions of the content. All authors read and approved the final manuscript.

## Pre-publication history

The pre-publication history for this paper can be accessed here:

http://www.biomedcentral.com/1471-227X/10/19/prepub
